# Neutral lipids associated with haemozoin mediate efficient and rapid β-haematin formation at physiological pH, temperature and ionic composition

**DOI:** 10.1186/1475-2875-11-337

**Published:** 2012-10-08

**Authors:** Melvin A Ambele, Timothy J Egan

**Affiliations:** 1 Department of Chemistry, University of Cape Town, Private Bag, Rondebosch, 7701, South Africa

**Keywords:** Haemozoin, β-haematin, Neutral lipids, Kinetics, Rates, Autofluorescence

## Abstract

**Background:**

The malaria parasite disposes of host-derived ferrihaem (iron(III)protoporphyrin IX, Fe(III)PPIX) by conversion to crystalline haemozoin in close association with neutral lipids. Lipids mediate synthetic haemozoin (β-haematin) formation very efficiently. However, the effect on reaction rates of concentrations of lipid, Fe(III)PPIX and physiologically relevant ions and biomolecules are unknown.

**Methods:**

Lipid emulsions containing Fe(III)PPIX were prepared in aqueous medium (pH 4.8, 37°C) to mediate β-haematin formation. The reaction was quenched at various times and free Fe(III)PPIX measured colorimetrically as a pyridine complex and the kinetics and yields analysed. Products were also characterized by FTIR, TEM and electron diffraction. Autofluorescence was also used to monitor β-haematin formation by confocal microscopy.

**Results:**

At fixed Fe(III)PPIX concentration, β-haematin yields remained constant with decreasing lipid concentration until a cut-off ratio was reached whereupon efficiency decreased dramatically. For the haemozoin-associated neutral lipid blend (NLB) and monopalmitoylglycerol (MPG), this occurred below a lipid/Fe(III)PPIX (L/H) ratio of 0.54. Rate constants were found to increase with L/H ratio above the cut-off. At 16 μM MPG, Fe(III)PPIX concentration could be raised until the L/H ratio reached the same ratio before a sudden decline in yield was observed. MPG-mediated β-haematin formation was relatively insensitive to biologically relevant cations (Na^+^, K^+^, Mg^2+^, Ca^2+^), or anions (H_2_PO_4_^−^, HCO_3_^−^, ATP, 2,3-diphosphoglycerate, glutathione). Confocal microscopy demonstrated β-haematin formation occurs in association with the lipid particles.

**Conclusions:**

Kinetics of β-haematin formation have shown that haemozoin-associated neutral lipids alone are capable of mediating β-haematin formation at adequate rates under physiologically realistic conditions of ion concentrations to account for haemozoin formation.

## Background

The malaria parasite, as well as a number of other blood-feeding organisms including the helminth *Schistosoma mansoni* and insects such as the kissing bug *Rhodnius prolixus*, digest large quantities of haemoglobin and have solved the problem of detoxifying haem by a common mechanism. They remove haem from solution in its oxidized ferric state (Fe(III)PPIX) by converting it to a highly insoluble solid known as haemozoin [1-3]. In *Plasmodium* it is widely accepted that this process is inhibited by important anti-malarials, particularly the 4-aminoquinolines chloroquine and amodiaquine, and possibly also quinoline and aryl methanols such as quinine and lumefantrine [4,5]. In addition, inhibition of the synthetic counterpart of haemozoin, β-haematin, has been used in several high-throughput screening studies to identify potential new anti-malarial chemotypes [6-9]. The mechanism of formation of haemozoin is thus of considerable interest.

It is now well established that haemozoin is closely associated with lipids in *Plasmodium*, *Schistosoma* and *Rhodnius*[10-13]. In the case of the last, crystals of haemozoin are associated with lipid bilayer-bound vesicles [12], but in the case of the first two organisms the crystals are associated with neutral lipid droplet-like structures [11-13]. In *Plasmodium falciparum* early crystals have been directly observed enveloped in lipid structures that have been dubbed lipid nanospheres. The lipids associated with haemozoin isolated by sucrose cushion centrifugation consist of a mixture of approximately 4:2:1:1:1 monostearoylglycerol (MSG), monopalmitoylglycerol (MPG), 1,3-dioleoylglycerol (DOG), 1,3-dipalmitoylglycerol (DPG) and 1,3-dilineoylglycerol (DLG) respectively [11].

Several studies have shown that β-haematin formation occurs rapidly in the presence of neutral lipids including *rac-*1-monomyristoylglycerol, the individual lipids associated with haemozoin referred to above and also the 4:2:1:1:1 blend of these lipids [11,14-16]. When hydroxo-Fe(III)PPIX dissolved in 0.1 M NaOH is premixed with the lipids in a water miscible solvent mixture (9:1 acetone-methanol) and carefully layered on top of an aqueous solution buffered at pH 4.8, a value close to that of the digestive vacuole (DV) of *P. falciparum* (the locus of haemozoin formation in the parasite), β-haematin is formed in high yield within minutes at 37°C [14-16]. It has been demonstrated that diffusion of acetone and methanol into the aqueous layer, which dilutes these solvents to low concentrations, creates a lipid emulsion in the aqueous medium. Confocal microscopy with the lipid-specific fluorescent dye Nile Red has shown that the neutral lipids as well as the neutral lipid blend (NLB) of 4:2:1:1:1 MSG/MPG/DOG/DPG/DLG all give rise to non-hollow lipid particles and that β-haematin formation occurs in close association with these artificial neutral lipid “droplets”, with an appearance strikingly similar to those seen in *P. falciparum* and *S. mansoni*[15]. Nile Red quenching has shown that Fe(III)PPIX rapidly partitions into these droplets if introduced into the aqueous medium. While rates of reaction are similar in the various lipids, there are marked differences in activation energy, with the more fluid unsaturated lipids exhibiting lower activation energy barriers and the NLB showing the lowest activation barrier of all [16]. This model system has thus provided a method for probing the mechanism of haemozoin formation under conditions that mimic the DV of the malaria parasite.

Despite two recent detailed investigations of β-haematin formation in neutral lipid emulsions [15,16], certain important questions remain to be addressed regarding the kinetics of lipid-mediated β-haematin formation. In particular, effects of lipid and Fe(III)PPIX concentrations on the rate of formation have not been probed. Nor has the question been investigated of whether ions and other molecules, that are likely to be present in the DV at appreciable concentrations, affect the kinetics. A detailed study of the effects of lipid and Fe(III)PPIX concentration, aqueous buffer identity, important simple physiological cations (Na^+^, K^+^, Ca^2+^, Mg^2+^), simple anions (HCO_3_^−^, H_2_PO_3_^−^) and the low molecular weight anionic metabolites, adenosine triphosphate (ATP), 2,3-diphosphoglycerate (2,3-DPG) and glutathione on β-haematin formation in the presence of the constituent lipids associated with haemozoin in *P. falciparum* under biomimetic conditions has therefore been undertaken.

## Methods

Porcine haemin (Cl-Fe(III)PPIX) was from Fluka (98%). All lipids and other reagents were obtained from Sigma-Aldrich (Vorna Valley, South Africa). Solutions of haematin (HO-Fe(III)PPIX) were prepared by dissolving 2 mg of Cl-Fe(III)PPIX in 0.400 ml of 0.1 M NaOH. These solutions were vortexed and sonicated for 3 – 5 min and then were made up to 1 ml with a 1:9 v/v mixture of acetone/methanol. Lipid solutions (3.31 mM) were prepared by dissolving MPG, MSG, DOG, DPG, DLG or NLB in 1:9 v/v acetone/methanol. Citric buffer was prepared at 50 mM concentration from citric acid, pH adjusted to 4.8 with NaOH. Acetate and MES buffers (50 mM, pH 4.8) were prepared from anhydrous sodium acetate and 2-(*N*-morpholino)ethanesulfonic acid (MES) sodium salt by pH adjustment with perchloric acid.

Kinetics of β-haematin formation were performed following methods previously reported [15,16]. Briefly, this was as follows: 50 ml of citric buffer (pH 4.8, 50 mM) was pre-incubated for 30 min in a water bath at 37°C in a 9 cm internal diameter Schott-Duran crystallization dish. HO-Fe(III)PPIX solution (0.5 ml) in acetone/methanol (1:9) was mixed with 1.0 ml of 1:9 acetone/methanol (control) or a lipid solution (3.31 mM) in the same solvent mixture. For studying the effect of lipid to Fe(III)PPIX ratio, the lipid solution was diluted with acetone/methanol to the desired ratio before mixing with Fe(III)PPIX. The resulting mixture was carefully layered on the surface of the pre-incubated citric acid using a 1 ml syringe. Incubation was allowed to proceed for varying lengths of time from 1 to 60 min. After the given incubation time, the solution was centrifuged at 10,000 rpm for 15 min. The supernatant, which contained no measureable Fe(III)PPIX concentration, was dicarded and the pellet washed with 1 ml of 5% pyridine prepared by mixing 5 ml pyridine, 50 ml acetone, 10 ml HEPES (0.2 M, pH 7.5) and 35 ml of water. Pyridine forms a low-spin complex with the Fe(III) centre in free Fe(III)PPIX which has an absorption maximum at 405 nm, but does not react with β-haematin under these conditions and is a particularly reliable method of quantitation [17]. The yield of β-haematin formed was obtained by measuring the absorbance of the supernatant from the pyridine washed pellet at 405 nm wavelength using a Varian Cary 100 UV–VIS spectrophotometer following dilution of 0.05 ml of the solution into 1 ml of water. The absorbance provides a measure of the unreacted Fe(III)PPIX remaining at each given time point. Percent conversion to β-haematin was then obtained by difference, using the control as the measure of 0% conversion. Data were fitted to an exponential equation, in keeping with previous studies using lipids [14-16].

To study the effect of lipid:Fe(III)PPIX ratio at fixed lipid concentration, the same procedure described above was adopted, but the concentration of Fe(III)PPIX dissolved in acetone/methanol was increased using a fixed lipid concentration. The effect of buffers on kinetics was investigated by replacing the aqueous citric buffer with the same volume of either actetate buffer or MES (both 50 mM at pH 4.8). To observe the effect of ions and other cellular components on kinetics of β-haematin formation, these substances were included in the buffer. This involved dissolution of NaCl, Na_2_HPO_4_, NaHCO_3_, KCl, adenosine 5’-triphosphate disodium salt or glutathione in the citric buffer solution prior to pH adjustment. Each of these salts was added at both red blood cell cytoplasmic and serum concentrations. Glutathione was prepared in argon purged solutions and the reaction performed under an Ar atmosphere. For Ca^2+^ and Mg^2+^, CaCl_2_·2H_2_O and MgCl_2_·6H_2_O were prepared in the same way, but acetate buffer was used instead of citric buffer because of the potential of citrate to coordinate these alkaline earth metal ions.

Yields of β-haematin were determined as described above for kinetics experiments, except that samples were incubated for 30 min and a reading taken at the end of the experiment to determine the conversion to product. The effect of 2,3-DPG on β-haematin formation was confined to its effect on the overall yield because of the fact that this reagent is not available in quantities needed for kinetics experiments. For this purpose, 2,3-diphospho-D-glyceric acid pentasodium salt was dissolved in the citric buffer prior to pH adjustment.

For Fourier transform infrared (FTIR) spectroscopic and transmission electron microscopic (TEM) characterization of β-haematin, samples were prepared using the method described above and allowed to incubate for 10 min. Material was collected just below the surface of the solution (in the mixing zone formed between the acetone/methanol lipid solution and the citrate buffer). This is clearly visible as a narrow milky emulsion layer at the boundry between the dark Fe(III)PPIX-containing layer and the clear underlying buffer solution.

For FTIR the product was dried over P_4_O_10_ in a dessicator. The dried solid was gently crushed to a fine powder using a mortar and pestle. The finely ground powder was mixed with Nujol to form a Nujol mull. The Nujol mull was then used for FTIR spectroscopy to obtain a spectrum of the product using a Perkin Elmer Spectrum 100 FT-IR spectrophotometer.

For TEM, materials collected from the various preparations of β-haematin, from the same boundary layer as described for FTIR experiments, were deposited on a carbon coated grid. The grid was stained for 5 min with uranyl acetate to improve contrast and immediately washed with distilled water. The grid was allowed to dry before viewing using a TECNAI G^2^ transmission electron microscope.

Confocal microscopy was performed at various time points during the formation of β-haematin. Again, samples were collected as described for FTIR and TEM experiments. For these studies, a 2 μl suspension of the materials was placed on a glass microscope slide. A thin glass coverslip was carefully placed on top of the solution to avoid air bubble formation in the solution between the two slides. The slide was then mounted inverted onto a LSM510-META Zeiss confocal microscope for fluorescence imaging. The excitation wavelength was at 516 nm and emission was imaged between 575 and 630 nm.

## Results

### Yields of β-haematin formation as a function of lipid concentration

**Figure 1:**
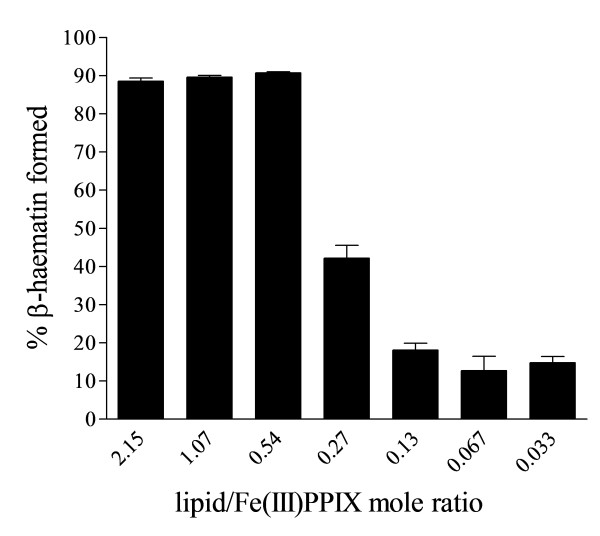
**β-haematin formation with NLB gave a high yield at higher lipid/Fe(III)PPIX mol ratios.** The high yield stays relatively constant from 2.15 L/H (64.3 μM total lipid) to 0.54 L/H after which the yield suddenly drops to a value of about 10% below 0.13 L/H. Citric buffer (50 mM, pH 4.8), 37°C, 30 min incubation. Constant total Fe(III)PPIX concentration of 30 μM. Error bars represent SEM (L/H = 2.15 and 1.07, n = 6; L/H = 0.54 and 0.27, n = 8; L/H = 0.13 and 0.067, n = 4; L/H = 0.033, n = 2).

**Figure 2:**
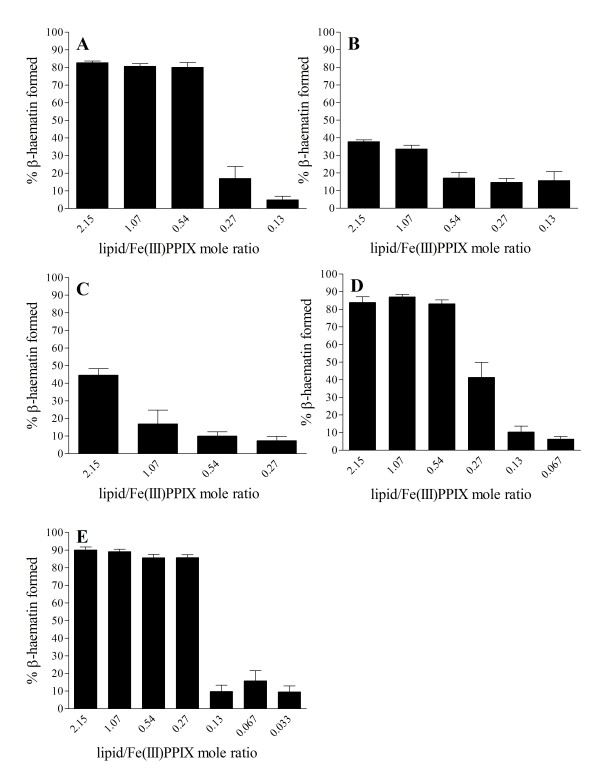
**Yields of β-haematin formed in the presence of individual constituent lipids of NLB vary with the identity of the lipid.** High yields of about 60–80% were obtained with MPG at L/H ratios from 2.15 to 0.54 with a sharp decrease in yield to less than 10% below 0.27 L/H (**A**). MSG and DPG at 2.15 L/H gave lower yields of about 40%, which decrease sharply to about 10–20% at L/H ratios of 0.54 and 1.07 respectively (**B** and **C**). DLG gave high yields around 80–90% at higher mol ratios (2.15–0.54) which decreases sharply to about 40% at 0.27 L/H mol ratio and drops further to about 10% and lower at 0.13 L/H (**D**). DOG appears to be the most efficient lipid with a high yield of almost 90% between 2.15 L/H and 0.27 L/H before decreasing sharply between 0.27 and 0.13 L/H (**E**). Citric buffer (50 mM, pH 4.8), 37°C, incubated for 30 min. Constant total Fe(III)PPIX concentration of 30 μM. Error bars represent SEM (A, C – E, n = 3; B, n = 4).

An apparent overall concentration of NLB of 64.3 μM (total lipid), corresponding to a 2.15 lipid:Fe(III)PPIX (L/H) mol ratio, gave a high yield of β-haematin in the region of 90%. (Note that the lipid forms an emulsion, so this concentration refers to the number of micromoles of lipid per litre of emulsion.) When the concentration of Fe(III)PPIX was kept constant, the yield did not change markedly until the lipid decreased below a mol ratio of 0.5. Thereafter the yield fell sharply before levelling out at very low values (Figure 1). Looking at the yields of β-haematin formed with the different constituent lipids of the NLB, a similar pattern was obtained with MPG as shown in Figure 2A. MSG and DPG on the other hand, gave much lower yields, close to 40%, even at the highest concentration of the lipid used in this study (64.3 μM), but similar yields to NLB were obtained at the lower lipid concentrations (Figure 2B and 2C). The low yields at high lipid concentrations might be a result of the lower solubility of these two lipids. Indeed, some precipitated lipid was evident in these solutions. This hypothesis is strongly supported by the observation made with DPG that increasing the temperature to 60°C, which solubilises the lipid, results in an increase in yield at 0.54 and higher L/H to almost 90% and gives an overall pattern of behaviour almost identical to MPG. The unsaturated lipids DLG and DOG gave higher yields of β-haematin (80 – 90%) at 37°C, similar to NLB and MPG at higher lipid concentrations. Interestingly, DLG still had a yield of about 40% at 0.27 L/H mol ratio (Figure 2D) and DOG appears to be even more efficient as it gave a much higher yield of β-haematin of about 80% at a 0.27 L/H mol ratio (Figure 2E) and only started to decrease below this concentration. The mol ratio below which the sharp decrease in yield is observed is hereafter referred to as the cut-off ratio. In the case of NLB and MPG this corresponds to an approximate value of 0.54 L/H.

### Effect of Fe(III)PPIX concentration on β-haematin yield

**Figure 3:**
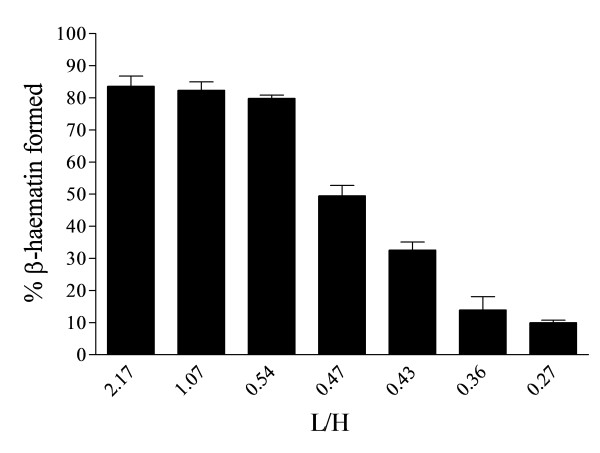
**Yields of about 80% β-haematin were obtained with 7.4 to 30 μM Fe(III)PPIX and 16.1 μM MPG (2.17 to 0.54 L/H ratio).** The yield started to decline from 34 to 37 μM Fe(III)PPIX (0.47–0.43 L/H) to about 30–50% and levels out to a lower yield of about 10% at 45 μM (0.36 L/H) and below. Incubation time was 30 min, citric buffer (50 mM, pH 4.8), 37°C. Error bars represent SEM (n = 3).

The concentration of MPG corresponding to the cut-off ratio of 0.54 L/H in Figure 2A (16.1 μM) was used to study the effect of Fe(III)PPIX concentration on the β-haematin yield. Starting from a Fe(III)PPIX concentration of 7.44 μM in experiments otherwise identical to those described above, the concentration of Fe(III)PPIX was increased while keeping that of MPG constant. The β-haematin yield was found to stay relatively constant to 30 μM Fe(III)PPIX, then declined before levelling out at 45 μM with a yield of about 10% (Figure 3), giving a cut-off ratio identical to those seen when the ratio is altered by decreasing the lipid concentration at constant Fe(III)PPIX concentration.

### Effects of lipid identity and concentration on kinetics of β-haematin formation

**Figure 4:**
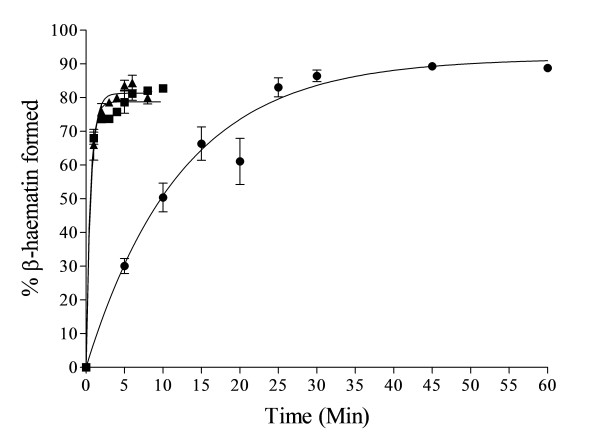
**Fast kinetics were observed with NLB at higher mol ratios of 2.15 (■) and 1.07 L/H (▲).** These reactions were complete within 10 min with yields of about 80% and conform to first-order kinetics. The rate constants were 1.9 ± 0.2 and 1.6 ± 0.1 min^−1^ respectively. Relatively slower kinetics were obtained at 0.54 L/H mol ratio (●), with *k* = 0.082 ± 0.006 min^−1^. Citric buffer (50 mM, pH 4.8), 37°C. Constant total Fe(III)PPIX concentration of 30 μM. Error bars represent SEM (n = 4).

In accord with previous studies [16], the kinetics of β-haematin formation mediated by NLB were found to be fast at mol ratios of 2.15 L/H and 1.07 L/H (64.3 and 32.2 μM total lipid). These reactions were complete within 10 min at yields of about 80%. On the other hand, the kinetics at 0.54 L/H were considerably slower but the reaction gave a similar final yield of about 80% when allowed to go to completion, which occurred within about 1 hr (Figure 4).

**Figure 5:**
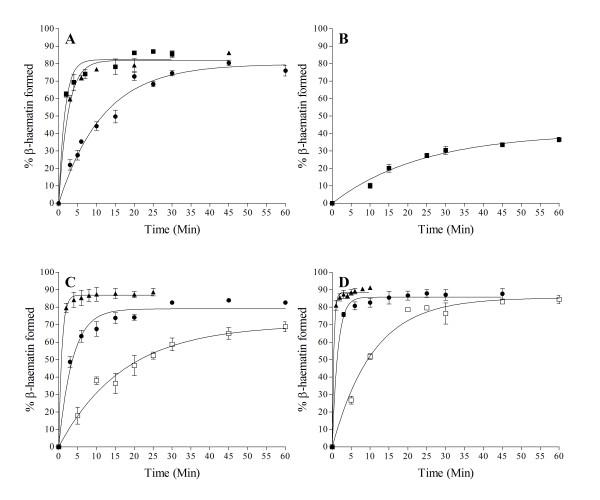
**MPG exhibited fast kinetics at 2.15 L/H (■) and 1.07 L/H mol ratio (▲) with*k*** **= 0.61 ± 0.07 and 0.40 ± 0.03 min**^**−1**^**respectively, while at 0.54 L/H mol ratio (●), the reaction is much slower with*k*** **= 0.085 ± 0.006 min**^**−1**^**, similar to that of NLB (A).** The reaction with MSG at the highest lipid concentration (2.15 L/H) is slower than NLB and MPG at 0.54 L/H mol ratio, with *k* = 0.044 ± 0.007 min^−1^ (**B**). Kinetics with DLG at 1.07 L/H (▲) and 0.54 L/H (●) appear to be faster than that of NLB at same mol ratio with *k* = 1.2 ± 0.2 and 0.27 ± 0.02 min^−1^ respectively, while at 0.27 L/H mol ratio (□) the reaction was slower with *k* = 0.058 ± 0.009 min^−1^ (**C**). DOG mediated exceptionally fast kinetics at 1.07 L/H (▲) and 0.54 L/H mol ratio (●) with *k* = 2.4 ± 0.2 and 0.69 ± 0.08 min^−1^ respectively, while the kinetics at 0.27 L/H (□) were slower with *k* = 0.094 ± 0.008 min^−1^, comparable to NLB and MPG at the mol ratio of 0.54 L/H (**D**). Citric buffer (50 mM, pH 4.8), 37°C. Constant total Fe(III)PPIX concentration of 30 μM. Error bars represent SEM (n = 4, except D for which n = 3).

Of the constituent lipids of the NLB, kinetics of the reaction mediated by MPG most closely resembled those seen with NLB. The reaction was fast at 2.15 L/H and 1.07 L/H mol ratios, reaching completion within less than 10 min. The slower reaction at 0.54 L/H mol ratio was comparable to the NLB at same mol ratio (Figure 5A). By contrast, MSG at 2.15 L/H mol ratio mediates relatively slow kinetics (Figure 5B) with a much lower yield of about 35% after 45 min. Compared to NLB and MPG, the reaction is slower even than that observed at a the lower mol ratio of 0.54 L/H. The kinetics mediated by DLG at 1.07 and 0.54 L/H mol ratios are faster than NLB and MPG at the same 0.54 L/H mol ratio (Figure 5C), indicating that this lipid on its own promotes β-haematin formation faster than the NLB at same concentration. DOG, which has been shown above to be very efficient in forming high yields of β-haematin, also exhibits the fastest kinetics at all the L/H mol ratios (Figure 5D). The kinetics of β-haematin formation with DOG at 0.27 L/H mol ratio can be compared to NLB and MPG at 0.54 L/H mol ratio.

The similarity in kinetic data at 0.54 L/H mol ratio for both the NLB and MPG (Figures 4 and 5A), with almost the same rate constants, suggest that MPG is a good model lipid for the NLB. The unsaturated diglyceride components of the NLB (DLG and DOG) are not available in large quantities, are prohibitively expensive and are unstable towards oxidation. For these reasons, MPG was chosen to further study the effect of ions on the kinetics of β-haematin formation.

Finally, an interesting observation is that the diameter of the vessel in which the experiments were performed affects the kinetics. This is almost certainly the result either of the characteristics of lipid droplets formed, which probably depend on the rate of mixing of the lipid solution with the aqueous buffer, or efficiency of transport of Fe(III)PPIX to the lipid droplets, which probably depends on the thickness of the mixing layer. Thus, the use of a 100 ml beaker with a 5 cm diameter, instead of the usual 9 cm Schott-Duran crystallization dish, resulted in a decrease in rate constant to 0.042 ± 0.005 min^−1^, a reduction of 50%. All other experiments were thus conducted in 9 cm Schott-Duran crystallization dishes.

### Effect of lipid to Fe(III)PPIX ratio on kinetics of β-haematin formation below the cut-off ratio

**Figure 6:**
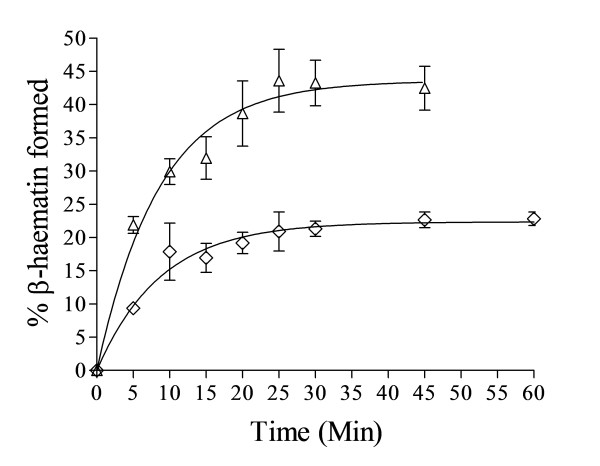
**Kinetics of β-haematin formation at ratios of 0.46 L/H (Δ) with*k*** **= 0.12 ± 0.02 and 0.36 L/H (**⋄**) with*k*** **= 0.11 ± 0.02 min**^**−1**^**respectively, are comparable to that at 0.54 L/H (where*k*** **= 0.085 ± 0.006 min**^**−1**^**).** Citric buffer (50 mM, pH 4.8), 37°C, MPG. Total Fe(III)PPIX concentrations were 35 and 45 μM respectively. Error bars represent SEM (n = 4).

In order to determine whether the sudden decrease in overall yield of β-haematin with decreasing lipid/Fe(III)PPIX ratio below the cut-off is a result of a decrease in rate of reaction, or a decrease in extent of reaction, the kinetics of the reaction were determined at two different ratios below the cut-off of L/H for MPG. The ratios were changed by increasing the quantity of Fe(III)PPIX relative to lipid. The results presented in Figure 6 show that there is little change in the half-lives for these apparent first-order processes. Hence this demonstrates that there is no further decrease in rate constants of the reaction. The dramatic decrease in yield below the cut-off is thus a result of decreased extent of reaction.

### Characterization of reaction products

Since the assay used to measure yield and reaction kinetics involves determination of leftover unreacted Fe(III)PPIX via formation of a low spin pyridine complex, these measurements do not directly confirm formation of β-haematin. Furthermore, since the measurements involve extraction of the unreacted Fe(III)PPIX from the reaction medium, it does not conclusively prove that the reaction happens *in situ* in the reaction medium. For these reasons additional experiments to characterise the reaction products using FTIR spectroscopy, TEM and electron diffraction were performed. Confocal microscopy was also used to demonstrate that the reaction actually occurs *in situ*.

**Figure 7:**
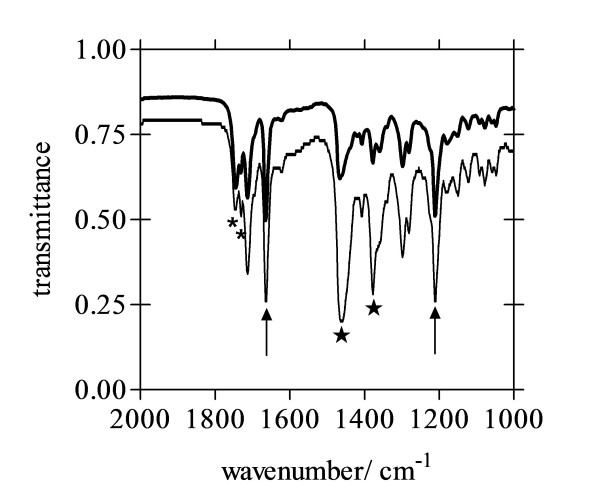
**FTIR spectra of dried product obtained as Nujol mulls.** Products were those formed using NLB (thick line) or MPG (thin line) and show characteristic β-haematin peaks at 1662 cm^-1^ and 1210 cm^-1^ (arrows). Both products gave an identical infrared spectrum. Nujol peaks (★) and contaminating lipid peaks (*) are marked. Reaction conditions: 2.15 L/H ratio (64.3 μM total lipid), citric buffer (50 mM, pH 4.8), 37°C, 10 min incubation.

Figure 7 shows FTIR spectra of reaction products obtained using NLB and MPG. Both exhibit strong infrared peaks at 1662 and 1210 cm^−1^. These are the characteristic peaks of β-haematin, arising from C=O and C−O stretching vibrations of the coordinated propionate groups in the μ-propionato dimers of Fe(III)PPIX that make up the structure of β-haematin. With the exception of peaks from contaminating lipids and nujol, the remaining peaks in the infrared spectrum are all identical to those found in spectra of β-haematin and haemozoin [18].

**Figure 8:**
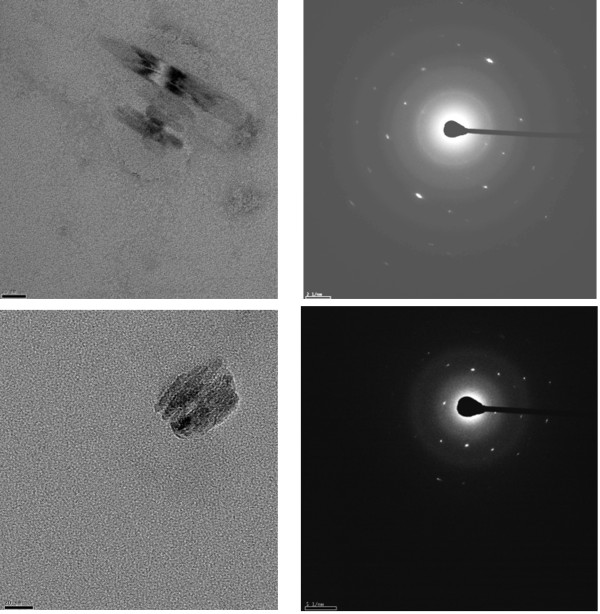
**TEM (left panels) and electron diffraction (right panels) of β-haematin obtained from an MPG mediated process (L/H = 0.54).** The particles are very small (scale bar = 20 nm), but show well defined diffraction spots conclusively confirming that the product is crystalline.

While FTIR spectra unequivocally demonstrate formation of the Fe(III)-propionate bond of β-haematin, it does not necessarily prove the formation of the crystalline product corresponding to haemozoin. To confirm the formation of such a product TEM images of the product obtained using MPG to mediate the process were recorded. TEM clearly shows the formation of an apparently crystalline product (Figure 8), although the crystals formed are very much smaller than natural haemozoin. To establish that the product is crystalline, electron diffraction measurements were made. These confirm that the product is indeed crystalline.

**Figure 9:**
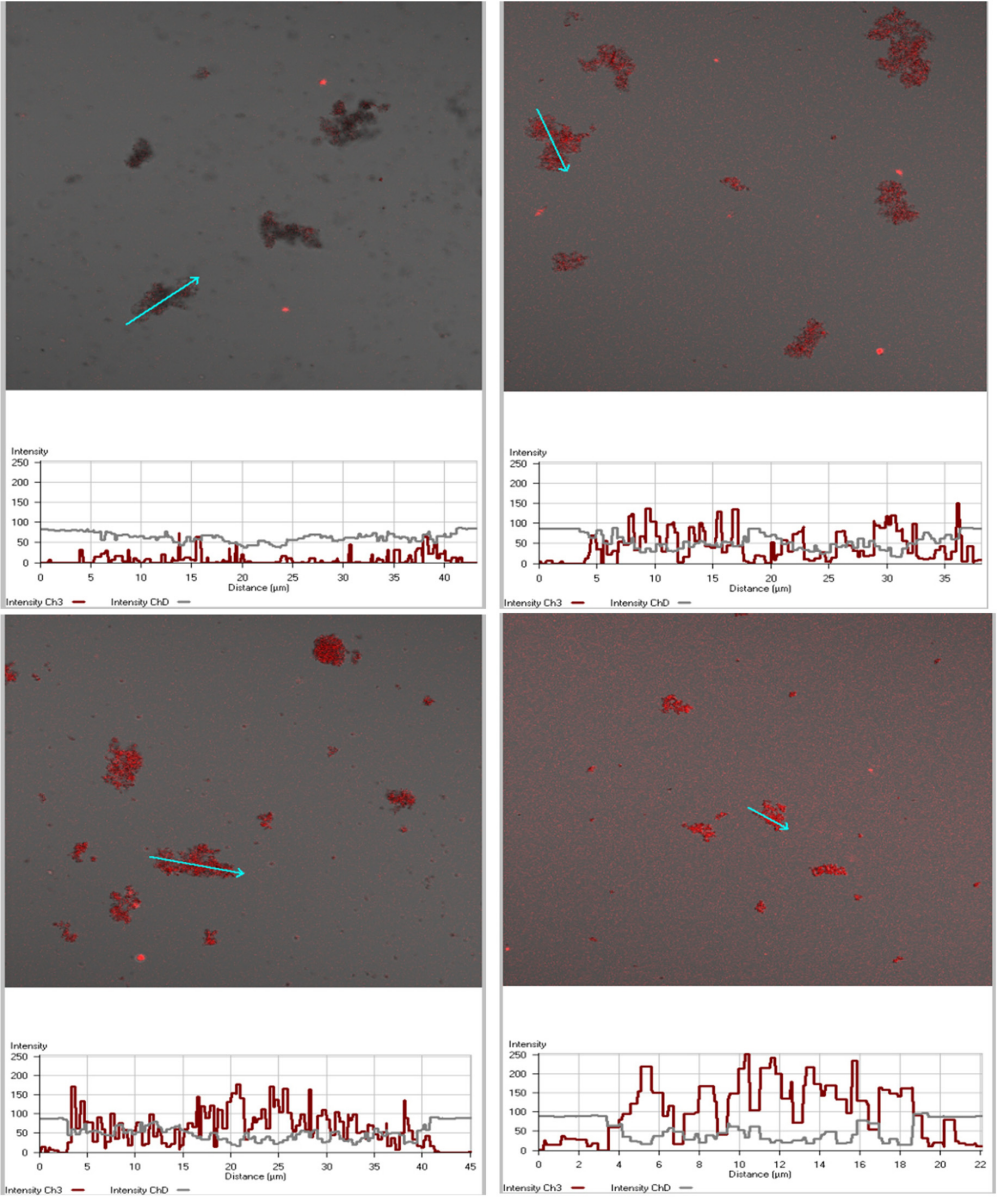
**Confocal laser microscopy images of MPG lipid aggregates in extracts taken at various time points during lipid mediated β-haematin formation.** Reaction times were 10 min (top left), 20 min (top right), 30 min (bottom left) and 60 min (bottom right). The dark non-fluorescent areas are unconverted Fe(III)PPIX, either precipitated, or lipid associated. Fluorescence profiles taken along cross-sections indicated by an arrow in each image are given below the images in red. The grey trace (ChD line) represents the transmitted light channel). Steady formation of β-haematin crystals is indicated by increasing fluorescence and is in qualitative agreement with kinetic traces, indicating about 60 min required to reach completion. Excitation was at 516 nm, emission in the range 575–630 nm. Reaction mediated by MPG, 0.54 L/H ratio, citric buffer (50 mM, pH 4.8), 37°C. Confocal microscopy was performed on wet samples. Acquisition conditions: λ_ex_ = 561 nm; λ_em_ = 575–630 nm; pinhole diameter 444 μm; output power 20% transmission; scan zoom 1.0; objective LD C-Apochromat 40×/1.1 W. No z-stacking.

While protoporphyrin IX is strongly fluorescent, the presence of the Fe centre in Fe(III)PPIX completely quenches this fluorescence. However, it is well established that haemozoin is autofluorescent, exhibiting an excitation maximum at 555 nm and emission maximum at 577 nm. This property has recently been shown to arise from an exciton recombination mechanism involving a Frankel-type exciton process [19]. The autofluorescence is thus dependent on the presence of the ordered crystal lattice of haemozoin or β-haematin. Indeed, it is present only in the crystalline product and it has been shown that even changes in hydration state of the lattice affect the process. Thus fully hydrated and fully dehydrated β-haematin exhibits this autofluorescence phenomenon, but partially hydrated material is non-fluorescent [19]. Autofluorescence thus provides a hitherto unexploited method for monitoring formation of β-haematin *in situ*. Figure 9 shows confocal microscope images of samples removed from the reaction mediated by MPG at a 0.54 L/H mol ratio at various times. It clearly shows an increase in β-haematin autofluorescence with time that arises exclusively within lipid droplets in the emulsion. The rate of growth of the fluorescence signal is in good qualitative agreement with the kinetic data described above. These data confirm that β-haematin formation occurs in situ and in association with the lipid. It should be noted that these images cannot be used to make a direct quantitative comparison with the kinetic data because factors such as aggregation of individual crystals, path length between the microscope slide and coverslip and evaporation of the solution on the slide may all affect fluorescence intensity.

### Effect of buffer

**Figure 10:**
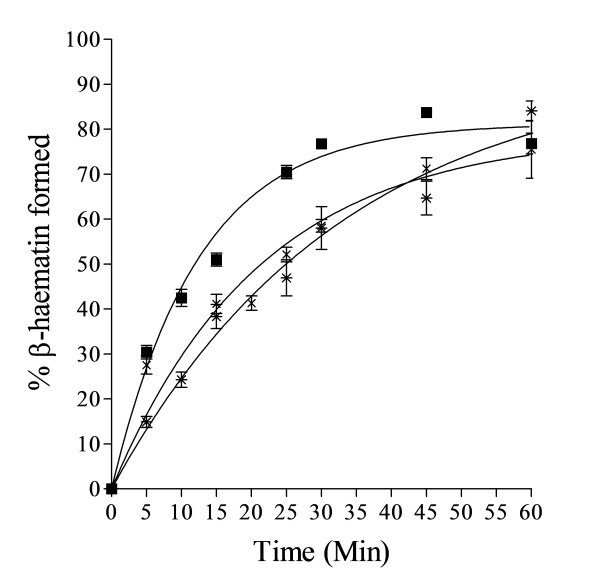
**Effect of buffer on the kinetics of β-haematin formation.** Rate constants obtained with citric (■), acetic (×) and MES buffers (*) were 0.080 ± 0.005, 0.047 ± 0.006 and 0.030 ± 0.004 min^-1^ respectively. Reaction mediated by MPG (0.54 L/H), pH 4.8 (50 mM buffer), 37°C. Constant total Fe(III)PPIX concentration of 30 μM. Error bars represent SEM (n = 4).

In the experiments described above, as well as in previous studies on rates of lipid mediated β-haematin formation, citric acid/sodium citrate was used to buffer the aqueous phase. In a previous study in which β-haematin was formed at a water-octanol interface it was shown that buffer identity (citric buffer versus MES) had no influence on reaction rate [14]. For that reason the effect of buffer on the reaction was investigated. As shown in Figure 10, the buffer identity has only relatively small effects on the reaction kinetics. Indeed, there is no significant change in rate constant when citric buffer is replaced with acetic acid/sodium acetate. On the other hand, there is a decrease in rate constant when MES buffer is used, but the change is only a little more than two-fold, but tending towards a higher final yield. Thus, effects of buffers appear to be minor.

### Effect of physiologically relevant ions and other biomolecules on kinetics

In addition to partially digested haemoglobin, the DV also contains various cations and anions that it obtains from the RBC, either as a result of ingestion of RBC cytoplasm, or through the action of ion pumps. Concentrations of ions in the DV are currently not known, but it is known that during the course of the blood cycle, the parasite modifies the RBC so as to markedly alter the RBC cytoplasm, which comes to more closely resemble the blood serum in its ionic content [20]. RBC cytoplasmic ion concentrations and serum ion concentrations would therefore probably represent two reasonable extrema for the possible ionic conditions in the DV. The effects of these ions at both values were investigated (Table 1). As shown in Table 1, ions at RBC cytoplasmic and serum concentrations have only relatively small effects on the rate constants of β-haematin formation mediated by MPG. The divalent Mg^2+^ and Ca^2+^ cations are the most active with respect to decreasing reaction rates. However, since they are present in fairly low concentrations, it is unlikely that they have much influence on reaction rates in the parasite. Similarly, phosphate has a marked effect in relation to its low concentration, but ATP seems to have no significant effect on reaction kinetics.

**Table 1:** **Effects of biologically relevant cations and anions on kinetics of β-haematin formation mediated by MPG (0.54 L/H), citric buffer (50 mM, pH 4.8), 37°C**^**a**^

**Added ion or biomolecule**	**Concentration (mM)**		***k*(min**^**−1**^**) ± SEM (n = 4)**
−		(control)	0.085 ± 0.006
Na^+^	8	RBC concentration	0.067 ± 0.006
Na^+^	150	serum concentration	0.031 ± 0.004
K^+^	150	RBC concentration	0.045 ± 0.005
Mg^2+^	1.75	RBC concentration	0.038 ± 0.005^f^
Mg^2+^	1.5	serum concentration	0.050 ± 0.005
Ca^2+^	3	serum concentration	0.030 ± 0.005^f^
H_2_PO_4_^−^	1	RBC concentration^b^	0.033 ± 0.004
HCO_3_^−^	15	RBC concentration^c^	0.026 ± 0.004
ATP	1.5	RBC concentration	0.039 ± 0.005
all ions combined	^d^	RBC concentrations	0.037 ± 0.006
all ions combined	^e^	serum concentrations	0.037 ± 0.003
glutathione	2.48	RBC concentration	0.024 ± 0.003^g^

^a^All ions were added to 50 mM citrate/sodium citrate buffer, which represents a background value. ^b^Total RBC phosphates. ^c^At pH 4.8 present as CO_2_. ^d^ 8 mM Na^+^, 150 mM K^+^, 1 mM H_2_PO_4_^−^, 15 mM HCO_3_^−^ and 1.5 mM ATP. ^e^ 150 mM Na^+^, 1.75 mM Mg^2+^ and 3 mM Ca^2+^. ^f^ 50 mM acetate/acetic acid buffer instead of citrate. ^g^ Under Ar, all solutions purged with Ar.

Finally, the effects of both 2,3-DPG and glutathione were examined. Since 2,3-DPG is available only in very small quantities, a full kinetic experiment was not feasible. Instead, a yield measurement was performed at 30 min, using MPG mediated β-haematin formation, 0.54 L/H, pH 4.8, 50 mM citric buffer at 37°C with 4.5 mM 2,3-DPG. The experiment was performed in triplicate and the yield was found to be 70.7 ± 1.9%, a value consistent with that obtained at the same time point for other anions (H_2_PO_4_^−^, HCO_3_^−^, ATP) and suggesting a similarly small effect of 2,3-DPG on the process. Glutathione is readily available and its effect on kinetics of the process was investigated. However, since it can generate reactive oxygen species through redox cycling in the presence of oxygen, the experiment was conducted under argon. A decrease in reaction rate constant was observed in the presence of 2.48 mM glutathione, but this was similar to that obtained with many of the other ions. This suggests that this concentration of glutathione, corresponding to that present in the RBC, is also unlikely to unduly interfere with lipid mediated haemozoin formation.

## Discussion

Formation of β-haematin mediated by NLB, MPG and the unsaturated diglycerides DLG and DOG is highly efficient, with conversions in the range 80 – 90%. These yields remain constant down to L/H ratios below 0.5 in the case of NLB and the monoglyceride MPG and almost 0.25 for the diglycerides DLG and DOG. This suggests that conversion remains high until the number of fatty acid chains per Fe(III)PPIX molecule drops below 0.5. Changes in yield as a function of L/H ratio could be envisaged to arise either from slower rates of conversion, or decreased extent of conversion, but with unchanged rates. The results of this study point to a combination of these two factors. Above the cut-off ratio, rate constants for conversion to β-haematin depend on the L/H ratio, with larger rate constants at higher lipid concentrations. The yield reaches a plateau simply because the reaction is effectively complete by the time the yield measurement is made after 30 min of incubation. The observed yield cut-off is thus to a large extent simply a result of the rate decreasing to the point where the reaction is no longer complete when the yield is measured. However, below the cut-off ratio, rate constants no longer decrease and the further decrease in yield appears to be a result of an actual decrease in extent of conversion. Importantly, all of the observed rate constants are greater than the lower limit previously reported to be required to account for the fact that conversion of Fe(III)PPIX to haemozoin occurs at an adequate rate for non-haemozoin haem to be undetectable in the malaria parasite by Mössbauer spectroscopy (0.017 min^−1^, corresponding to a half-life of 40 min) [14].

The observation that the maximal yield of β-haematin never reaches 100% can probably be ascribed to precipitation of a small portion of the Fe(III)PPIX before it partitions into the lipid. Previous studies suggest that such precipitated Fe(III)PPIX would convert slowly, if at all to β-haematin, since the rate of conversion of the solid is controlled by dissolution, rather than crystallization rates [21,22]. This probably also explains why yields drop sharply below the cut-off ratio without a further decrease in rate of conversion. Under these conditions there is simply too little lipid to maintain the Fe(III)PPIX in a dissolved state. As regards rates of conversion, these would also be expected to be controlled by the surface area of the lipid droplets, because at higher L/H ratios there is more lipid surface available to nucleate β-haematin.

Rate constants for conversion of Fe(III)PPIX to β-haematin are dependent on lipid identity. The trend DOG > DLG > MPG > MSG can be directly connected to the previously reported activation energy values measured using these lipids which follows the order DOG < DLG < MPG < MSG [16]. The lower activation energy observed for DOG and DLG compared to MPG and MSG has previously been ascribed to the greater fluidity of the diglycerides, which are in fact above their melting points at 37°C [16]. In addition, thermal analysis has shown that MPG and MSG exhibit two liquid crystalline forms, or polymorph, that are designated α and sub-α. The conversion from the α to sub-α polymorphs occurs at a lower temperature in MPG than in MSG, with the former below 37°C [16]. It is possible that this may play a role in producing the higher rate constant observed with MPG, which favours the α polymorph at 37°C. An alternative or additional factor that could account for different rates of conversion may be differences in the surface area to volume ratio of lipid particles resulting from size differences with different lipid types. A study on the effects of lipid particle size on β-haematin formation is currently underway.

The confocal microscopy observations form the first direct observation of β-haematin formed *in situ* in neutral lipid droplets and demonstrate the potential of the autofluorescence phenomenon for following β-haematin formation. Although qualitative in this study, these data do illustrate that the process occurs at a rate that is essentially commensurate with kinetics measured by conversion of extracted unreacted Fe(III)PPIX to a pyridine complex. Thus, the kinetic results are unlikely to have been unduly affected by the analytical method used and are probably a good representation of the process occurring in the lipid particles themselves. FTIR together with TEM observations confirm that the reaction product is indeed β-haematin and is crystalline in nature. However, the crystals are much smaller than haemozoin. Preliminary studies suggest that lipid particle size is a major factor in determining crystal size. The effects of lipid identity and lipid particle size on β-haematin crystals formed in the reaction are currently under detailed investigation.

The observation that MPG can be used as a convenient model for NLB together with the fact that the reaction rate can be considerably slowed without a decrease in overall yield by adjusting the L/H ratio has facilitated examination of the effects of buffers and ions on rates of conversion. In previous investigations, the rate of β-haematin formation was so fast that it would have been difficult to obtain sufficiently precise rate constants to examine these effects [15,16]. Using a 0.54 L/H ratio with MPG, the effects of buffer identity as well as those of a series of ions on the kinetics could be examined. Replacement of citric buffer with acetic acid/acetate has no effect on the kinetics, while replacement with MES causes a 2.8-fold increase in the half-life of the reaction, but an increased yield, so that overall conversion is almost the same during the first 60 min of reaction. Inclusion of various cations and anions at either RBC or serum concentrations also results in only relatively small changes. None cause more than a 3.3-fold increase in the half-life of the reaction at their respective concentrations. This effect is not increased by combining the ionic components, since neither a combination resembling the uninfected RBC cytoplasm, nor the serum causes more than a 2.3-fold increase in half-life of the process. Finally, the biological reducing agent glutathione also causes only a 3.5-fold increase in half-life at physiologically relevant concentration. This suggests that the biological ionic milieu is unlikely to have a major effect on the efficiency of lipid-mediated haemozoin formation.

Sullivan and co-authors reported a L/H ratio in sucrose cushion centrifugation extracts of haemozoin from *P. falciparum* of 0.15 [11]. Examination of Figure 1 shows that this ratio lies just at the bottom of the cut-off where poor conversion yield is observed with all of the lipids investigated. On the other hand, at least with MPG, the rate of conversion remains fast, reaching completion in less than 20 min at similar ratios. Given that the drop in yield is probably a result of the inability of the lipid to maintain the full quantity of Fe(III)PPIX in solution, this may not be an important factor in the biological milieu since it must be remembered that in the living organism free haem is released as a result of continuous digestion of haemoglobin. Thus, the process does not involve introduction of the full bolus of Fe(III)PPIX at the beginning of the reaction as was the case in the experiments conducted here. Development of a suitable method to mimic the entire proteolysis and haemozoin formation process would be required to establish whether the lipid alone is capable of mediating haemozoin formation, or whether other cellular constituents such as the recently proposed haem detoxification protein (HDP) [23], is required to ensure assembly of haemozoin in sufficient yield. Such an experiment would have the added benefit of allowing the effects of peptide fragments generated during globin degradation to be assessed, a factor which could not be assessed here.

## Conclusions

The findings of this study that NLB and MPG, but not MSG are highly efficient at converting Fe(III)PPIX to β-haematin with yields at or above 80% is in good agreement with previous work by Pisciotta *et al.*[11]. Furthermore, ions and other molecules likely to be present in the DV are unlikely to dramatically influence the reaction rate at physiological concentrations. While overall yield drops at physiologically relevant lipid to Fe(III)PPIX ratios, rates remain adequate to account for haemozoin formation. These observations strongly support the hypothesis that haemozoin formation in the malaria parasite is lipid mediated. Furthermore, confocal microscopy studies making use of β-haematin autofluorescence have directly demonstrated that β-haematin forms in situ in close association with the lipid component of the emulsion.

## Abbreviations

Cl-Fe(III)PPIX: Haemin; DLG: 1,3-dilineoylglycerol; DOG: 1,3-dioleoylglycerol; DPG: 1,3-dipalmitoylglycerol; DV: Digestive vacuole; Fe(III)PPIX: Iron(III)protoporphyrin IX; FTIR: Fourier transform infrared; HO-Fe(III)PPIX: Haematin; L/H ratio: Lipid to Fe(III)PPIX ratio; MES: 2-(*N*-morpholino)ethanesulfonic acid; MPG: Monopalmitoylglycerol; MSG: Monostearoylglycerol; NLB: Neutral lipid blend; TEM: Transmission electron microscopy.

## Competing interests

The authors declare that they have no competing interests.

## Authors' contributions

Both authors conceived and designed the study. MAA performed the experiments, data analyses and prepared the figures and tables. Both authors drafted the manuscript. Both authors read and approved the final manuscript.
